# Apoptosis in Hemocytes Induces a Shift in Effector Mechanisms in the *Drosophila* Immune System and Leads to a Pro-Inflammatory State

**DOI:** 10.1371/journal.pone.0136593

**Published:** 2015-08-31

**Authors:** Badrul Arefin, Lucie Kucerova, Robert Krautz, Holger Kranenburg, Farjana Parvin, Ulrich Theopold

**Affiliations:** 1 Department of Molecular Biosciences, Wenner-Gren Institute, Stockholm University, Stockholm, Sweden; 2 Institute of Neurobiology, University of Münster, Münster, Germany; Kansas State University, UNITED STATES

## Abstract

Apart from their role in cellular immunity via phagocytosis and encapsulation, *Drosophila* hemocytes release soluble factors such as antimicrobial peptides, and cytokines to induce humoral responses. In addition, they participate in coagulation and wounding, and in development. To assess their role during infection with entomopathogenic nematodes, we depleted plasmatocytes and crystal cells, the two classes of hemocytes present in naïve larvae by expressing proapoptotic proteins in order to produce hemocyte-free (Hml-apo, originally called Hemo^less^) larvae. Surprisingly, we found that Hml-apo larvae are still resistant to nematode infections. When further elucidating the immune status of Hml-apo larvae, we observe a shift in immune effector pathways including massive lamellocyte differentiation and induction of Toll- as well as repression of imd signaling. This leads to a pro-inflammatory state, characterized by the appearance of melanotic nodules in the hemolymph and to strong developmental defects including pupal lethality and leg defects in escapers. Further analysis suggests that most of the phenotypes we observe in Hml-apo larvae are alleviated by administration of antibiotics and by changing the food source indicating that they are mediated through the microbiota. Biochemical evidence identifies nitric oxide as a key phylogenetically conserved regulator in this process. Finally we show that the nitric oxide donor L-arginine similarly modifies the response against an early stage of tumor development in fly larvae.

## Introduction

Insect innate immune responses include reactions that depend primarily on the production of soluble mediators such as antimicrobial peptides that are secreted by the fat body and responses that require the recruitment and activation of immune cells (hemocytes [1]). Examples for the latter are phagocytosis, the encapsulation of large foreign objects and the formation of nodules, which entrap bacteria in case their numbers exceed the phagocytic capacity of hemocytes [1, 2]. Nevertheless, most immune reactions require a close collaboration between secreted fat body-derived and hemocyte factors [1]. In *Drosophila* two hemocyte types are present in naïve larvae: the phagocytic plasmatocytes and crystal cells, which harbor key factors of the phenoloxidase-activating system (PAS) including prophenoloxidase (proPO) itself [3]. Activated phenoloxidase produces cytotoxic intermediates, and contributes to crosslinking during wound healing and immune reactions [4]. Ultimately the PAS leads to the production of melanin at wound scabs, capsules and nodules. Lamellocytes, a third class of hemocytes, are rarely observed in naïve larvae but differentiate upon encapsulation and to a lesser extent after wounding [3].

To dissect the respective contributions of the fat body versus hemocytes, ablation of hemocytes has been performed [5–7]. Subsequent infection of larvae and adult flies revealed the hemocytes’ contribution to immune responses. Ablation of hemocytes was achieved by expressing proapoptotic proteins alone or in combination in hemocytes using the hemocyte-specific Hemolectin (*hml*) driver, which is primarily expressed in plasmatocytes and crystal cells. The results from these studies confirm the importance of hemocyte phagocytosis and their contribution to the induction of antimicrobial peptides [5, 6]. Using a similar strategy the induction of the Toll pathway was shown to depend on the hemocyte-derived cytokine Spätzle [7]. Ablation of hemocytes allowed normal development up to the pupal stage but eclosion rates for adult flies were lower than in controls. However, it was unclear what caused pupal lethality [5–7].

During the last larval instar melanotic spots were observed in one study where hemocytes were deleted [5] but not in the others [6, 7]. Together with the appearance of melanotic spots a population of enlarged non-phagocytic cells was observed which was proposed to consist of non-functional plasmatocytes or abnormal lamellocytes [5]. Here we wished to assess the effects of apoptotic ablation of hemocytes and its effects in a nematode infection model that involves hemocyte recruitment and activity. Additional rationales included our observation that apoptosis can be observed during formation of a hemolymph clot [8] and that the apoptotic marker phosphatidylserine enhances PO activity [9]. Both findings suggest that apoptotic markers are exposed during normal physiological processes where they may regulate downstream activities that are important for immune responses. We find that hemocyte-specific induction of apoptosis induces a shift in the immune status of larvae characterized by the appearance of melanotic spots, lamellocyte differentiation, induction of the Toll pathway and developmental defects. Most of the effects are ameliorated by antibiotic treatment or upon changing the culture medium and appear to be modulated by nitric oxide levels. Similarly we observe that in a *Drosophila* pro-tumor model, feeding a nitric oxide donor leads to an increase in lamellocyte numbers and the appearance of melanotic spots.

## Experimental Procedures

### Drosophila Stocks

Fly stocks were maintained under standard conditions. Crosses were performed at both 25° and 29°C (see figure legend for specific information). To induce apoptosis in hemocytes, we used the protocol described previously [5, 6]. The following stocks were obtained as generous gifts: *hml* ^*Delta*^-Gal4,UAS-*eGFP* (II), *he*-Gal4 (III) and *he*-Gal4,UAS-*GFP*.*nls* (III) from Dan Hultmark, UAS-*hid*/*CyO Tb* (II) from Julian Royet, and UAS-*p35* (II) from Mitch Dushays lab. The *CG7607* RNAi line (v9208) was obtained from the Vienna *Drosophila* RNAi Center [10]. *Bx* ^*MS1096*^-Gal4 (II), UAS-*Ras* ^*V12*^ (III), UAS-*Rho1*.*V14* (III) and *w* ^*1118*^ were obtained from the Bloomington stock center. The following stocks were generated *Lz*-Gal4; UAS-*mCD8*::*GFP* (I; III)[11], and all UAS-*grim* lines [12].

### Nematode infections

Nematode infections were performed according to the protocol described before [13, 14]. Briefly, 62–68 h staged *Drosophila* larvae were washed in 25°C tap water and each larva placed in one well (96 well plate) containing a nematode suspension (25 nematodes in 10 μl). The plate was then sealed with parafilm and kept at 25°C. Mortality was scored 48 h after infection. Three replicates each containing 48 larvae and at least independent three biological replicates were analyzed. Entomopathogenic nematodes (EPN) of the species *Heterorhabditis bacteriophora* (strain H222 isolated from Pouzdrany, Czech Republic) were used for the infection.

### Immunohistochemistry

Larvae were dissected in 0.01 M phosphate-buffered saline (PBS; pH 7.4), fixed in 4% paraformaldehyde (PFA) in 0.1 M sodium phosphate buffer overnight in 4°C, washed three times for 10 minutes with 0.1% Triton-X in PBS and subsequently blocked in PBS containing 1% BSA and 0.1% Triton-X (PAT). Primary antibody was diluted in PAT to the recommended concentration. Incubation with primary antibodies was performed overnight at 4°C with gentle agitation followed by rinsing in PBS containing 0.1% BSA and 0.3% Triton-X (PBT) and incubation with secondary antibodies (diluted in PBT) overnight in 4°C. Nuclei were stained with Hoechst 33342 or DAPI. After three washes in PBT, guts or lymph glands were mounted in Fluoromount-G (SouthernBiotech).

The following antibodies and dyes were used: mouse monoclonal anti-Hemese (1:10 dilution, [15]) mouse monoclonal anti-L2 (1:50 dilution, [15]) mouse monoclonal anti-L1a,b,c (1:10 dilution, [15]); mouse monoclonal anti-Nimrod C1 (P1, 1:10 dilution, [15]), AlexaFluor 546-conjugated anti-mouse (1:1000 dilution, Invitrogen), DAPI (1:1000 dilution, Sigma-Aldrich), Hoechst 33342 (1:1000 dilution, Immunochemistry).

### Hemocyte preparation and counting

Third instar larvae were washed with tap water with gentle brushing, and then briefly cleaned with 70% ethanol followed by rinsing with PBS. Gentle brushing dislocated the resident (sessile) hemocytes releasing them into circulation [16]. Subsequently larvae were transferred and dissected immediately one in each well (in Hendley-Essex 12 multispot slides) containing 30 μl Schneider medium. In order to prevent PAS activation, Schneider medium containing anti-coagulant phenylthiourea (PTU) was used. For fixation, 4% PFA (paraformaldehyde) was used for 10 min. Hemocytes were subsequently washed with PBS and processed for microscopy or for further staining. For hemocyte counts, pictures taken with 20x objective (area- 430 X 328 μm) were analyzed with ImageJ or lamellocyte counts determined manually.

### Quantitative PCR

Total RNA extraction from third instar *Drosophila* larvae was performed as described previously [14]. The quality and concentration of the RNA were determined with a NanoDrop 2000 spectrophometer (Thermo Scientific). 1,000 ng of total RNA was applied for reverse transcription using SuperScript III Reverse Transcriptase (Invitrogen) and oligo(dT) (20-mer). qPCR was performed using the KAPA PROBE FAST Universal qPCR Master Mix (Kapa Biosystems) and the Custom TaqMan Expression Assays (Applied Biosystems) for *Drosomycin*, *Diptericin* and *Cecropin A1* [17]. The amplification was carried out in a Rotor-Gene Q (Qiagen). Each sample was analyzed in triplicate. Relative mRNA levels were normalized to *rp49* expression and standardized to the control genotype. The results are presented as the mean log2-transformed fold changes in transcript levels ± the standard deviations of the mean of at least 3 independent biological replicates.

### Microscopy

A Leica MZ FLIII fluorescence stereomicroscope associated with a Panasonic DMC-G2 camera was used to visualize larvae and adults, melanotic spots in transparent larvae and hemocyte recruitment. Visualizing hemocytes and melanotic masses from larvae was performed with a Hamamatsu ORCA-ER camera (C4742-95) coupled to a Zeiss Axioplan 2 microscope. Confocal images of dissected guts and lymph glands stained with different antibodies and Hoechst were taken with a Zeiss LSM 780 microscope.

### Antibiotic treatment, feeding Drosophila Instant medium (DIM) and arginine, and NOS inhibition assays

Experiments were performed at 29°C with standard fly food (SF) unless mentioned otherwise. For antibiotic treatment, each vial of fly food contained 150 μl antibiotic solution (5 mg/mL of ampicillin and kanamycin, [6]). For feeding DIM (Formula 4–24 Instant Drosophila Medium, Carolina), the manufacturer’s protocol was followed. For arginine feeding, larvae were reared on fly food containing 50 mM L-arginine. L-NAME (pharmacological inhibitor of NOS) and D-NAME were added to the fly food to a final concentration of 50 mM and 100–106 h staged larvae were transferred to the food.

### Statistics

Statistics for all nematode infection experiments were performed as described previously [14]. Statistics for quantification, and correlation studies were performed using Student’s t test (unpaired, two sided), Chi square test, and a Spearman Rank test respectively.

## Results

### Plasmatocytes and lamellocytes attach to nematode-inflicted wounds

**Fig 1:**
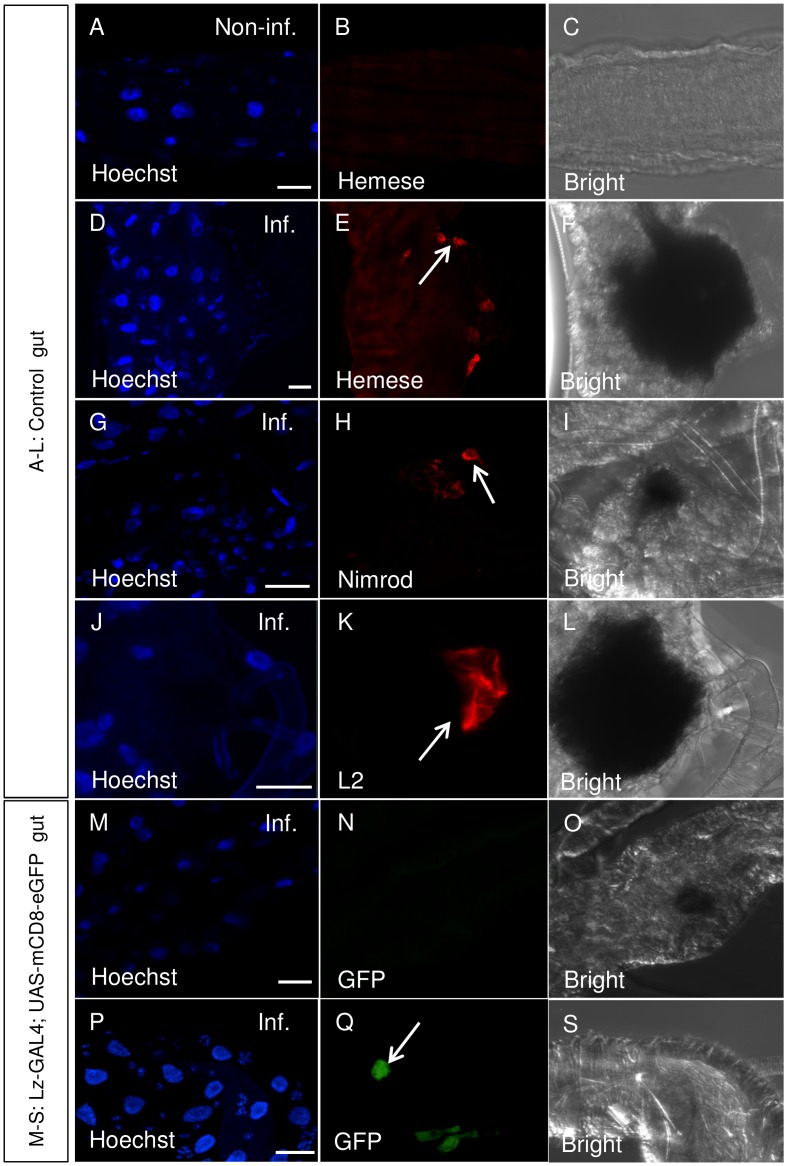
Plasmatocytes and lamellocytes attach to nematode-inflicted wounds. (A-L): Dissected gut from non-infected (A-C) and infected (D-L) late 3^rd^ instar vkg-GFP larva as control were visualized using DAPI (A, D, G, J, M, P,), pan hemocyte Hemese (B, E), plasmatocyte-specific Nimrod- (H) and lamellocyte-specific L2- (K) antibodies (the vkg-GFP, signal is not shown, but compare (14)). (B, E) Hemese staining was not observed in non-infected guts (B) whereas nematode-inflicted wounds showed Hemese, Nimrod and L2 staining (E, H, K). (N, Q) GFP-positive crystal cells (green) were not observed at the wound site (N), but they were detected at other places in the gut (Q). Bright field exposure (C, F, I, L, O and S) shows the extent of the wound due to melanization. Arrows indicate hemocytes, plasmatocytes, lamellocytes and crystal cells in E, H, K and Q respectively. Scale bars represent 20 μm.

*Drosophila* sessile hemocytes enter the circulation upon nematode infection (see S1 Fig) and [14]). Using a pan-hemocyte antibody (anti-Hemese) (Fig 1E) we detected hemocytes at the site of the wounds afflicted by nematodes during their passage of the midgut epithelium. No signal was observed in guts from non-infected larvae (Fig 1A–1C). We further used hemocyte subtype-specific antibodies and genetic tools (GFP expression in crystal cells) to determine which hemocyte subtypes were recruited to the wounds (Fig 1H, 1K and 1Q) and found that both plasmatocytes (Nimrod staining-Fig 1H) and lamellocytes (L2 staining Fig 1K) attached to wounds. In contrast, crystal cells while detected at other places of the gut were not found in the vicinity of the wound (compare Fig 1N with 1Q). Taken together this shows that hemocytes are recruited to the site of nematode entry into the hemocoel and adds to the evidence that cellular immunity is involved in controlling nematode infections.

### Hml-apo larvae are resistant to nematode infections

**Fig 2:**
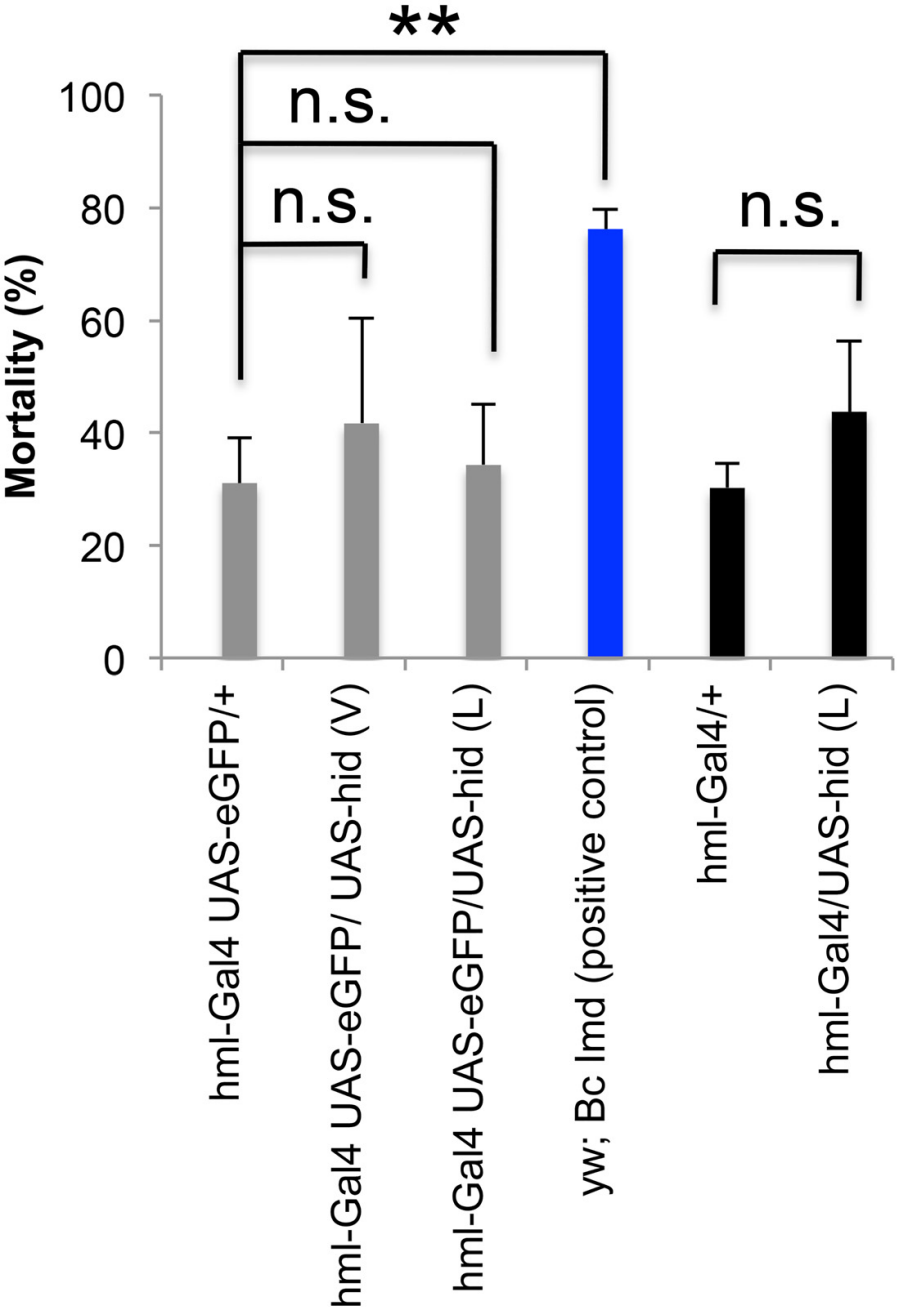
*Drosophila* larvae lacking hemocytes are not more susceptible to nematode infections. Hemocytes were depleted using two Hid insertion lines (viable-V or lethal-L) and two Hemolectin Gal4 driver lines. To visualize hemocytes, UAS-*eGFP* combined with *hml-*Gal4 was employed. *Hml*-Gal4,UAS-*eGFP* driven UAS-*hid* expression in both insertion lines successfully eliminated hemocytes (see S2 and S3 Figs). To maximize Hid expression we also used *hml*-Gal4 without UAS-*eGFP*. However, none of the crosses between Gal4 driver lines and Hid responder lines showed significantly increased mortality compared to the positive control (vx.Bc Imd see [13, 18]). The vertical axis shows normalized mortality and the negative control was set to 1. Data presented are means ± SD; t test: * p<0.05; **p<0.01.

To get a better understanding of the role hemocytes play during nematode infections, we depleted them in larvae using a previously established method that relies on expression of the pro-apoptotic protein Hid in combination with the hemocyte-specific Hemolectin driver (*hml* ^*Delta*^-Gal4, in the following abbreviated as *hml*-Gal4) [5, 6]. We also used a second pro-apoptotic protein (Grim). Initially the strength of two UAS-*hid* and four UAS-*grim* lines was tested (see S1 Table for details) and those with the strongest effects chosen for most of our subsequent experiments (UAS-*hid* (L) and UAS-*grim8*.*1* respectively). After expressing the two Hid lines in hemocytes using '*hml*-Gal4,UAS-*eGFP*' (abbreviated in the following as HFP), almost all hemocytes were GFP-negative, showing that apoptosis had been induced successfully in third instar larvae, adults (S2–S4 Figs) and already at the first larval instar (S5). To our surprise none of the crosses that led to hemocyte depletion showed significant changes in mortality upon nematode infection (Fig 2). To exclude a diluting effect on the transcriptional activator (Gal4) due to dual targeting of UAS-*eGFP* and UAS-*hid* we repeated the infection using *hml-*Gal4 without UAS-*eGFP* to maximize UAS-*hid* expression. However, this also did not increase mortality of the *Drosophila* larvae (Fig 2). Taken together, these observations suggest that counter to our expectation apoptotic hemocyte depletion in larvae did not increase their sensitivity towards nematode infections.

### Apoptosis in plasmatocytes and crystal cells triggers lamellocyte differentiation

**Fig 3:**
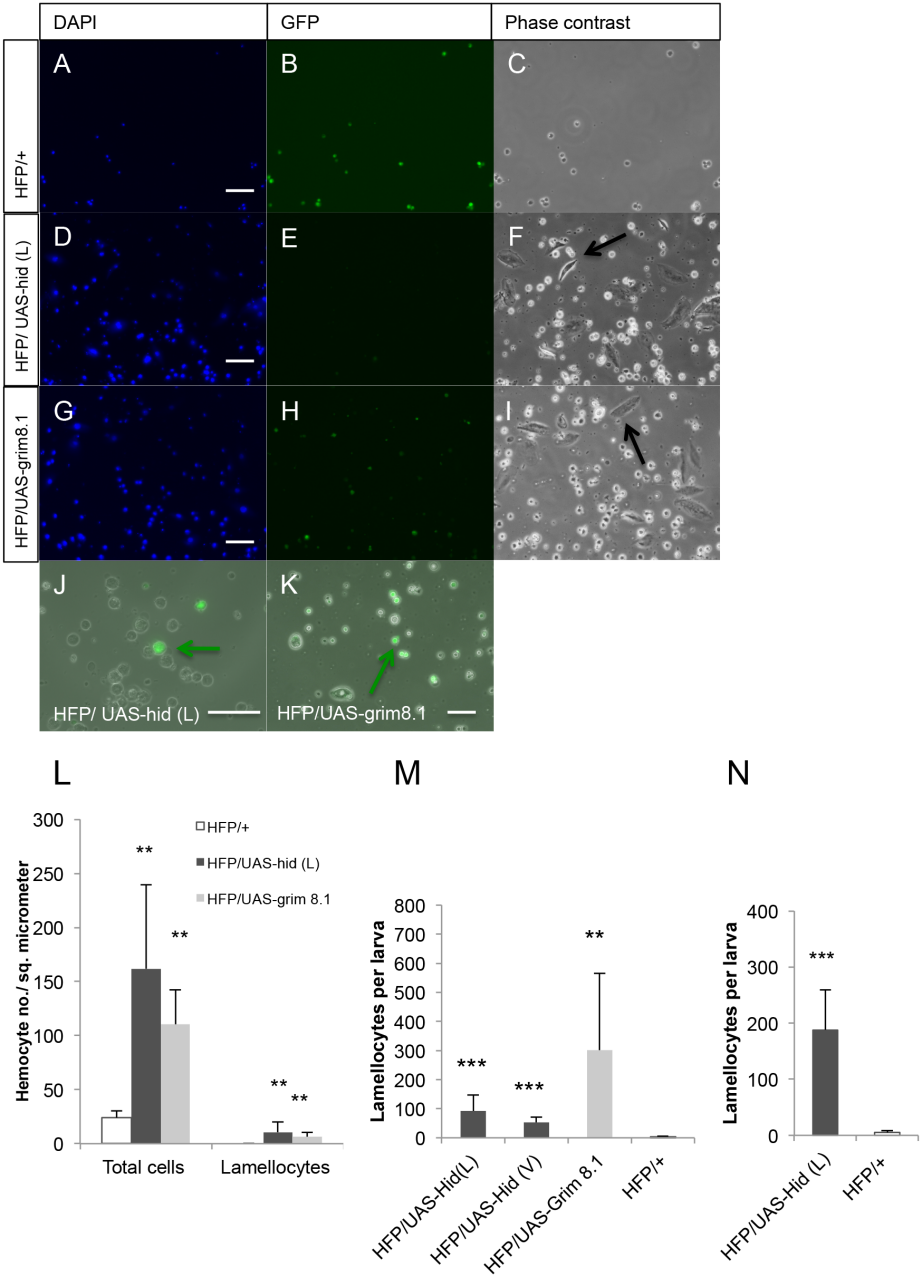
Induction of apoptosis in plasmatocytes and crystal cells triggers lamellocyte differentiation. Hemocyte preparations from 3^rd^ instar larvae were analyzed under the epi-fluorescence microscope. (A-I) Both Hid- and Grim-expressing samples showed massive lamellocyte differentiation (F and I) whereas control samples showed none (C). In addition when apoptotic cell bodies were included in the counts, an increase in counts was observed (using DAPI staining) in Hid- and Grim-expressing samples (D and G) compared to controls (A). (J, K) Some GFP-positive hemocytes were still detectable after Hid (J) or Grim (K) expression (the scale bars correspond to 50 μm). (L) Quantification of total cell (including apoptotic bodies) and lamellocyte numbers (hemocyte counts were determined within a defined area, see Material and Methods for details). Significantly higher numbers of total cells/cell fragments and lamellocytes were detected in Hid or Grim samples compared to controls. (M, N) Quantification of lamellocyte numbers per larva at 25° (M) and 29°C (N). Black arrow indicates lamellocytes (F and I), and green arrows indicate GFP-positive live hemocytes (J and K). HFP: *hml-*Gal4,UAS-*eGFP*. Data represent means ± SD; t test: **p<0.01, *** p<0.001.

To further investigate the effects of apoptosis in hemocytes, we examined hemocyte preparations from non-infected larvae where apoptosis had been induced. When we bled mid 3^rd^ to late 3^rd^ instar larvae after hemocyte-specific expression of Hid or Grim, GFP-positive hemocytes were drastically reduced in both lines (Fig 3E and 3H) compared to *hml-*Gal4,UAS-*eGFP/+* control crosses.

**Fig 4:**
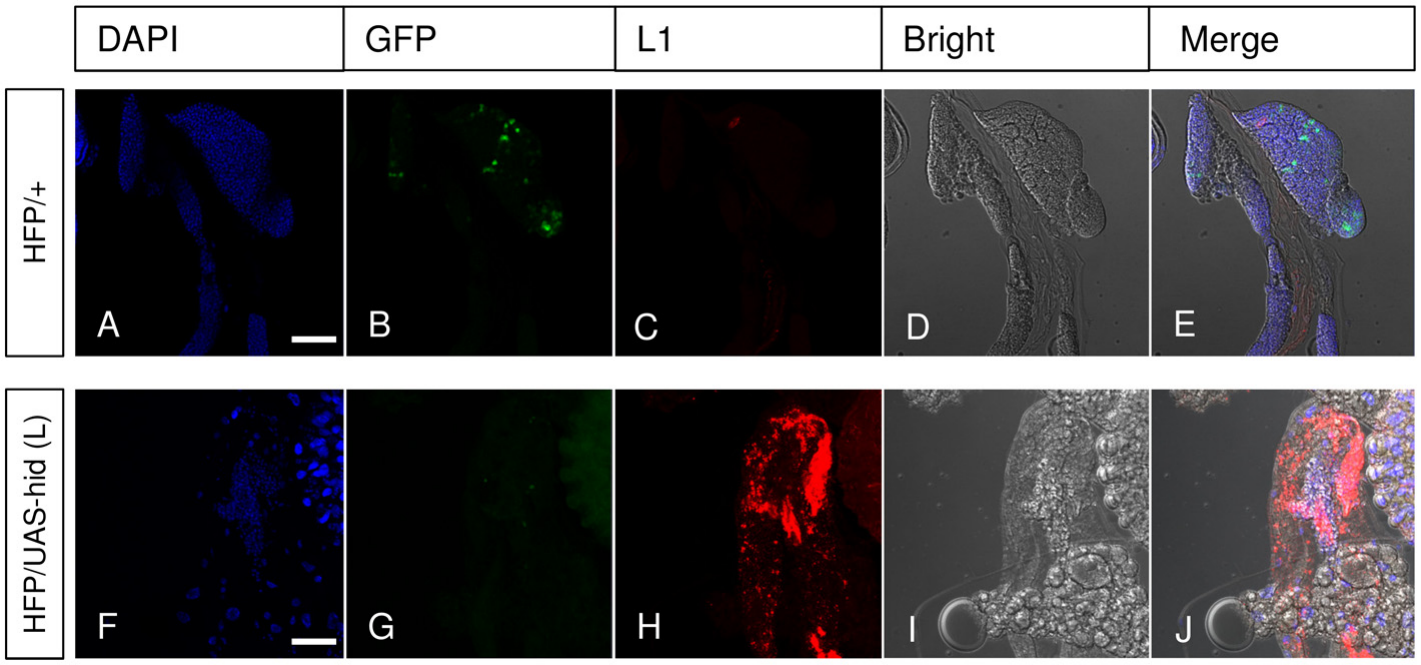
Lamellocyte differentiation in the lymph gland of Hml-apo larvae. Dissected fixed lymph glands from 3^rd^ instar larvae were stained with DAPI and the early lamellocyte-specific antibody L1. (B and G) Control (HFP/+) lymph glands show the presence of some plasmatocytes and/or crystal cells (B) whereas no GFP signal was observed in Hid-expressing lymph gland (G), which confirms effective elimination through apoptosis. Strong and extensive L1 staining was observed in the lymph gland of Hid-expressing larvae (H) whereas no L1 staining was found in the control (C). HFP: *hml-*Gal4,UAS-*eGFP*. The scale bars represent 50 μm.

Unexpectedly, we found massive differentiation of lamellocytes in both Grim- and Hid-expressing larvae (Fig 3F and 3I). Of note *hml-*Gal4,UAS-*eGFP* drives GFP transgene expression only in plasmatocytes and crystal cells and not in lamellocytes, which therefore are not targeted by expression of proapoptotic genes in our set-up [19]. Lamellocytes were already detected in the second and early third larval instar at a stage when the lymph gland still displayed a GFP signal indicating that depletion of gland-derived hemocytes had not occurred yet (S5) and is therefore likely due to the depletion of embryonic hemocytes. At the late third instar only few GFP-positive cells were left in both apoptotic lines (Fig 3J and 3K). When apoptotic cell bodies were included, total counts were found significantly increased in both Hid and Grim over-expressing larvae compared to control crosses (Fig 3L). This indicates that prior to their apoptotic death, the pool of plasmatocytes might have expanded. Alternatively, although we only included large DAPI-positive fragments in the counts, the fragmentation of apoptotic cells may lead to an overestimation of cell numbers. Increased lamellocyte titers were found both at 25°C and further increased at 29°C, the optimal temperature for the Gal4 system (Fig 3M and 3N). To detect lamellocyte differentiation in the lymph gland, we stained the glands using an early lamellocyte-specific antibody (L1) (Fig 4). Lymph glands from Hid over-expressing larvae showed substantial accumulation of L1 staining compared to the controls, suggesting that lamellocyte differentiation had occurred. In conclusion, these data suggest that plasmatocyte depletion under these conditions had been largely effective but appears to have induced a differentiation of lamellocytes. These findings confirm previous observations where lamellocyte-like cells had been reported after depletion of plasmatocytes with the *hml-*Gal4 driver [6].

### Melanotic masses appear after apoptotic depletion of hemocytes

**Fig 5:**
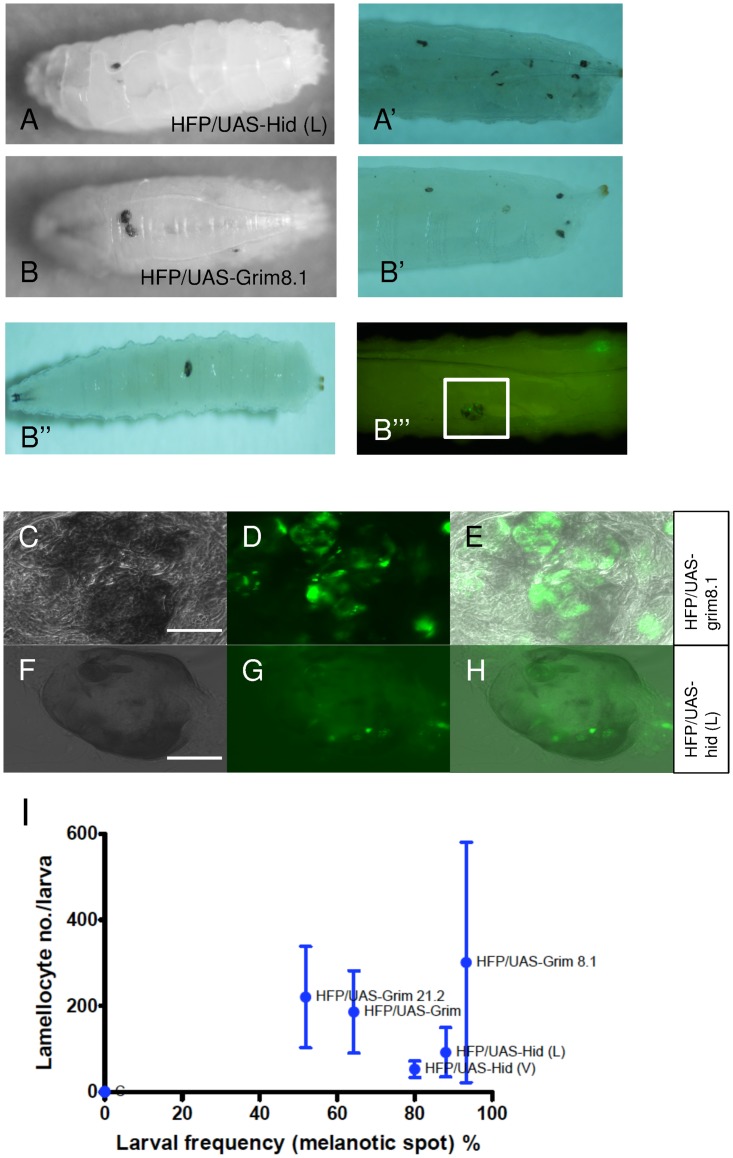
Melanotic masses are formed in Hid- and Grim-expressing larvae. (A-B’’’) Different patterns of melanotic masses were found in both Hid- (A-A’) and Grim-expressing larvae (B-B’’’). (C-E) The melanotic mass of the area marked in B’’’ was visualized at higher magnification (C-E). GFP-positive hemocytes were observed within the melanotic mass (D) indicating hemocyte origin. (F-H) Melanotic masses from Hid-expressing larvae also displayed a GFP signal (G) as in (D). (G). Of note, Hid expression was found stronger than Grim. (I) Positive correlation between larval frequency (melanotic spot) and lamellocyte numbers in different Hid and Grim lines (Spearman correlation, P value = 0.0108).

We observed melanotic masses or pseudo-tumors in Hml-apo larvae at late 3^rd^ instar larval stages leading to different patterns (Fig 5A and 5B”). These melanotic aggregates were found in both the anterior and posterior part of the larva and in circulation. To test whether melanization targets any particular organ, we dissected larvae but did not observe any organ-specific pattern. Instead melanotic masses appeared loosely attached to the fore- and hindgut. When they were analyzed microscopically (Fig 5C–5H), some GFP-positive cells were detected indicating that they were at least partially of hemocyte origin. When plotted against lamellocyte numbers the frequency of the melanotic aggregates clearly showed a positive correlation (Fig 5I) (P value = 0.0067) indicating that melanization might depend on lamellocyte phenoloxidase (PPO3) or that lamellocyte differentiation and melanization are co-regulated.

### The Toll pathway is activated and imd signaling is suppressed in Hml-apo larvae

**Fig 6:**
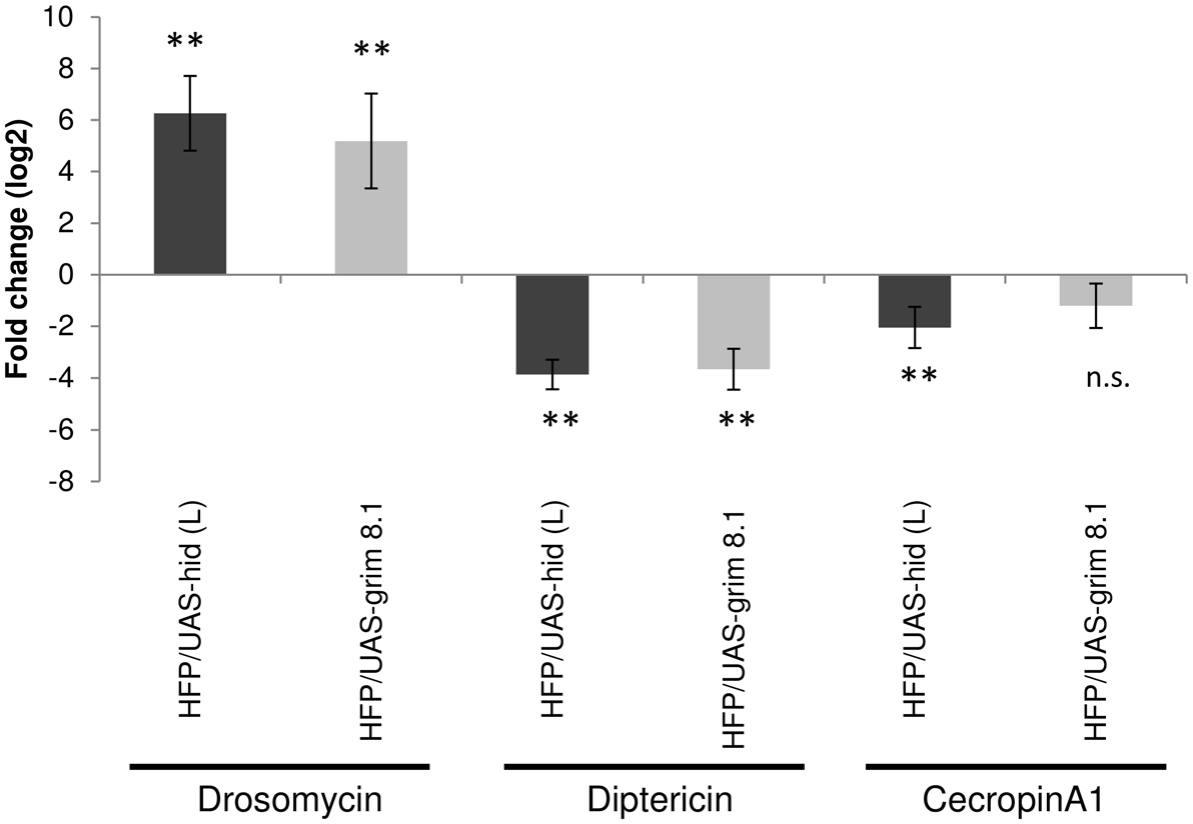
Constitutive Drosomycin expression signaling is up- and imd-dependent expression is down-regulated in hemocyte depleted larvae. Compared to controls, induction of *Drosomycin* was observed in both Hid- and Grim-expressing larvae whereas imd dependent *Diptericin* was downregulated. *CecropinA1*, which is regulated with input from both pathways (Toll and imd) showed an intermediate pattern although this was non-significant in Grim-expressing lines. The relative expression level (the ratio of pro-apoptotic samples/control) is shown as log2 mean from at least 3 independent triplicates ± SD; t test: * p<0.05; **p<0.01.

We next asked whether the development of melanotic masses correlated with an induction of the Toll pathway as suggested by earlier studies [20–22]. Quantitative PCR (q-PCR) using probes for *Drosomycin*, *Diptericin* and *CecropinA1* showed an upregulation of Toll-dependent *Drosomycin* whereas *Diptericin* was suppressed. *CecropinA1* levels were reduced in UAS-*hid* (L) over-expressing larvae whereas no significant changes were observed in the UAS-*grim8*.*1* over-expressing line (Fig 6).

**Fig 7:**
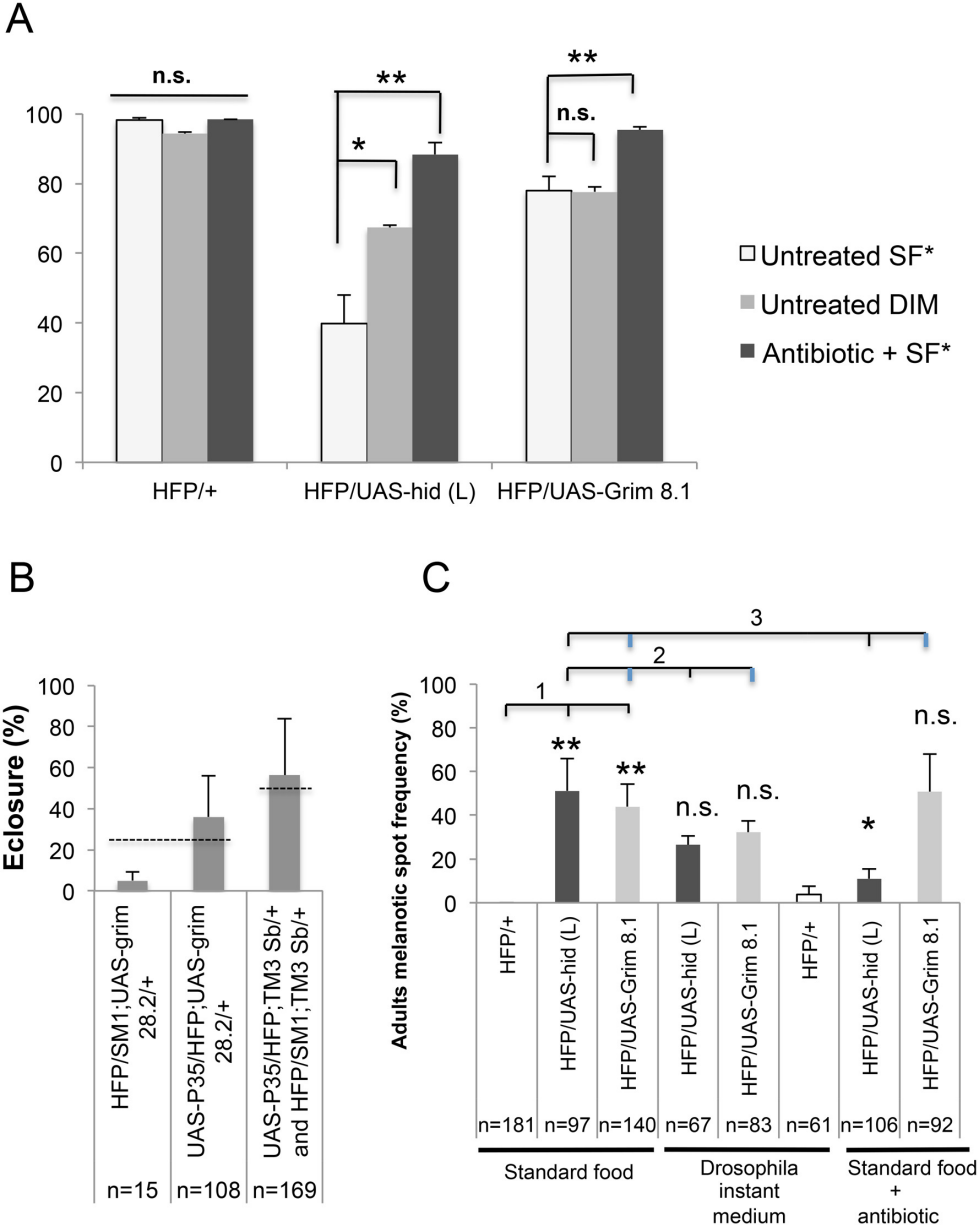
Antibiotic treatment, different fly medium and inhibition of apoptosis can rescue eclosion, and melanization defects after hemocyte depletion. (A) Both Hid and Grim lines showed significantly higher pupal lethality (measured as a drop in eclosure rate in %) than controls; antibiotic treatment rescued Hid and Grim induced lethality. Lethality of Hid-expressing larvae was also affected by using different fly media. SF—standard fly food (potato source). DIM—Drosophila instant medium (see S2 Table for the composition of the food, both parental lines were homozygous). (B) Coexpression of UAS-*grim28*.*2* with UAS-*p35* (caspase inhibitor) in the same larva rescued pupal lethality. Dashed lines indicate the expected frequency of eclosing flies for the crosses (25 and 50% respectively). (C) A higher melanotic spot frequency was found in adults in both Hid- and Grim-expressing lines compared to controls. Antibiotic treatment rescued melanotic mass formation in Hid- but not in Grim-expressing lines. The melanotic spot frequency was compared between Hid, Grim and controls using standard fly food (bracket 1), the influence of the food source (bracket 2, no significant differences) and the antibiotic treatment, (bracket 3, significant only for Hid-expressing larvae). Data presented are means ± SD; t test: * p<0.05; **p<0.01.

Furthermore, we also observed a high incidence of pupal lethality measured by a drop in eclosure rates of adult flies. Treatment with antibiotics rescued pupal lethality in both Hid and Grim over-expressing larvae (Fig 7A). Together with the previous observation (Fig 6) this indicates that the reduction in constitutive *imd* signaling or in *imd* inducibility had led to a loss of microbial control, which affected normal development. Similarly the food source influences eclosure rates after Hid-dependent hemocyte depletion most likely also by influencing the composition of the microbiome (Fig 7A, and [23]). Rescue of eclosure rates by co-expression of anti-apoptotic p35 confirms that it depends on the apoptotic effects of Hid and Grim on hemocytes ((Fig 7B). Although not to the same extent, the formation of melanotic spots was also influenced by administration of antibiotics to Hid-expressing larvae and by changing the food source, (Fig 7C) while Grim-expression had no significant influence. Taken together, these data show that hemocyte depletion led to a change in the immune status and made larvae more susceptible to environmental changes and changes in the microbial environment thus affecting normal development. Our data also identifies a reduction in imd signaling as a likely proximate cause of the pupal lethality.

### Elevated nitric oxide in Hml-apo larvae produces defective legs in adults

**Fig 8:**
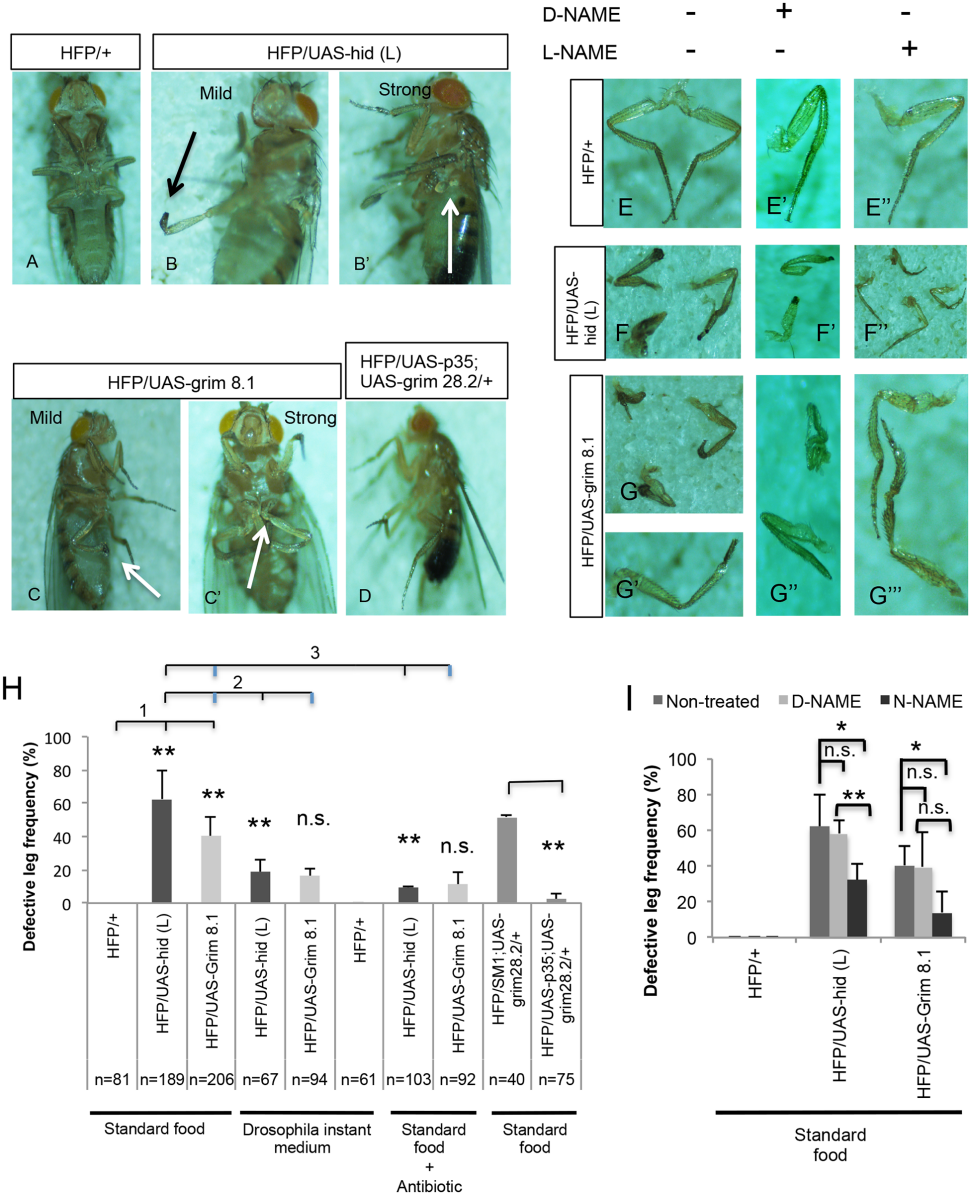
Hml-apo flies show a defective leg phenotype, which can be rescued by blocking apoptosis, pharmacological inhibition of NOS and antibiotic treatment. (A) Control adults (HFP/+) where legs were normal. (B-C’) Both Hid- and Grim-expressing adults showed defective legs ranging from shortened leg segments (mild phenotype, B and C) to the complete absence of a leg (strong phenotype, B’ and C’). In both cases, phenotypes were most pronounced for the 3^rd^ leg pair. Arrows indicate the defective leg phenotype (B, B’, C and C’). (D) Inhibiting apoptosis by co-expressing UAS-*grim28*.*2* with UAS-*p35* rescued the defective leg phenotype. (E-G’’’) shows isolated legs including (E) normal control adult legs (E, HFP/+) and defective legs in both Hid and Grim lines (F, G, and G’) in non-treated conditions. (E”) Feeding L-NAME (a pharmacological inhibitor of NOS) to 3^rd^ instar larvae, rescued the leg defects in both Hid and Grim adult flies (F” and G’’’) while feeding D-NAME (inactive isomer of NAME) did not (F’ and G”). (H) Quantification of the defective leg phenotype in apoptotic adults, and after rescue with antibiotic treatment and upon co-expressing UAS-*grim28*.*2* with UAS-*p35*. Defective legs were found in both Hid and Grim lines. Hid lines showed a stronger phenotype (such as a complete absence of legs, bracket 1) and a higher frequency of defective legs than Grim adults. Using a different food source (DIM) rescued the defective leg phenotype in Hid- but not Grim-expressing flies (bracket 2) and the same was true for antibiotics treatment (bracket 3). Blocking apoptosis using UAS-*p35* also rescued the defective leg phenotype (right part). (I) Quantification of defective leg penetrance after treatment (D-NAME and L-NAME) compared to non-treated flies. Data presented are means ± SD; t test: * p<0.05; **p<0.01 (n = 81, 120 and 62 for controls, 189, 114 and 62 for Hid-expressing flies and 206, 120 and 72 for Grim-expressing flies).

When examining adults after eclosion, we observed defects that particularly affected the third pair of legs. Either tarsal segments were missing or in extreme cases the whole leg was affected (Fig 8B, 8B’, 8C and 8C’). These defective leg phenotypes could also be rescued either by co-expressing p35, by feeding antibiotics or using DIM. The antibiotic treatment suggests that the microbiota contributes even to this phenotype either directly or via its effects on hemocytes. Interestingly, defective legs had been observed upon ectopic expression of mouse nitric oxide synthase [24]. This prompted us to test whether NO might similarly contribute to the leg defects in our system and indeed feeding a pharmacological inhibitor of NOS (L-NAME) but not its less active enantiomer D-NAME rescued the leg defects (Fig 8F, 8G and 8I). Potential sources for NO include the gut epithelium and lamellocytes [25, 26]. Due to its developmental effects when fed to larvae, the influences of L-NAME on eclosure rates could not be tested with sufficient stringency [27]. Instead when we applied the nitric oxide donor L-arginine to control flies, lamellocyte levels increased although neither melanization nor the leg defects could be further enhanced (S6 Fig). Conversely, feeding L-NAME (but not D-NAME) to Hid-expressing larvae reduced lamellocyte counts (S7 Fig). Together this means that NO promotes lamellocyte differentiation and that lamellocytes may in turn further enhance the NO concentration [26]. This positive feedback might explain the substantial increase in lamellocytes counts we observe (Fig 3, see Discussion for further details).

### L-arginine enhances melanization and lamellocyte frequency in a pro-tumor model

**Fig 9:**
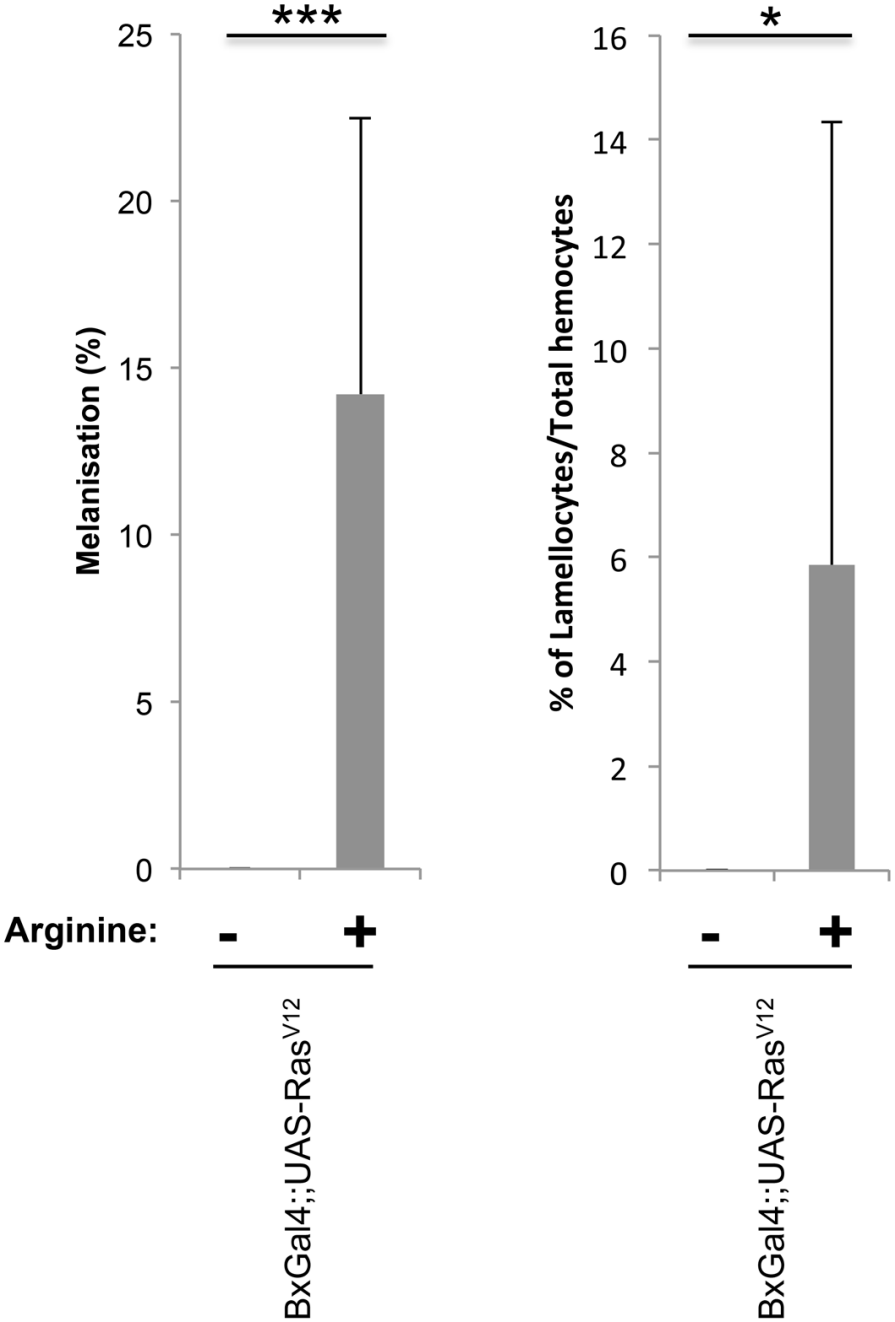
Standard fly medium supplemented with arginine (NOS substrate) increased melanization and lamellocytes in pro-tumor model. Dominant active Ras (Ras^V12^)-expressing larvae showed increased penetrance of melanization and lamellocyte formation (expressed as the relative frequency of lamellocytes) upon feeding the NO donor L-arginine.

Altogether the shift in immune effectors in particular the differentiation of lamellocytes is reminiscent of situations were immunity is chronically activated such as in a recently described tumor model [11]. In this system, expression of a dominant-active form of the Ras oncogene (Ras^V12^) induced apoptosis in the salivary glands. This attracted plasmatocytes, crystal cells and lamellocytes to the apoptotic tissue although without any detectable signs of melanization. Interestingly, when repeating the induction of a tumorigenic state in the presence of L-arginine in the medium, we found that both lamellocyte titers were increased and melanization was activated in a large fraction of the Ras^V12^-expressing larvae while without L-arginine neither of these changes was observed (Fig 9).

## Discussion

In this work we followed established protocols to deplete hemocytes with the goal to assess the contribution of hemocytes towards the immune response against nematodes. Hemocytes were successfully deleted by expressing proapoptotic proteins yet the effects on the susceptibility towards nematodes were at best moderate and non-significant. This prompted us to assess the effects of hemocyte depletion in more detail. We found that hemocyte depletion reproduced the previously reported reduction in eclosion rates of adult flies [5–7] and discovered a previously unnoticed effect on leg development in escapers. The drop in eclosion could be rescued by administering antibiotics confirming that it may be explained by a lack of keeping bacteria sufficiently restrained during metamorphosis [5–7]. This is likely due to a lack of phagocytosis after depletion of hemocytes. We observe additional changes in the immune system of hemocyte-depleted larvae. In general we detect more pronounced effects than previously noted [5]. These include differentiation of lamellocytes and the appearance of melanotic aggregates that either freely float in the hemolymph or attach to organs. In addition we found that in Hml-apo larvae constitutive Drosomycin expression is activated (through Toll or FoxO signaling, [28]) and constitutive imd signaling is reduced. This implies that hemocytes are required for a full activation of the imd pathway under naïve conditions in line with previous observations that the imd-dependent *Defensin* requires hemocyte activity for induction [29]. In addition to the lack of phagocytosis, imd downregulation may in fact contribute to the serious pupal defects we observe, by allowing faster septicemia due to bacterial release from the gut. Similar to the reduced eclosion rates, the frequency of melanotic aggregates and the defects in leg formation were rescued in the presence of antibiotics. Taken together this shows that apoptotic depletion of hemocytes induces compensatory changes in the immune system leading to developmental defects and that several of these effects depend on the presence of bacteria. On a technical note these findings indicate that when assessing the contribution of hemocytes to immunity based on depletion experiments, it may be advantageous to assess not only plasmatocyte depletion but also other immune parameters.

**Fig 10:**
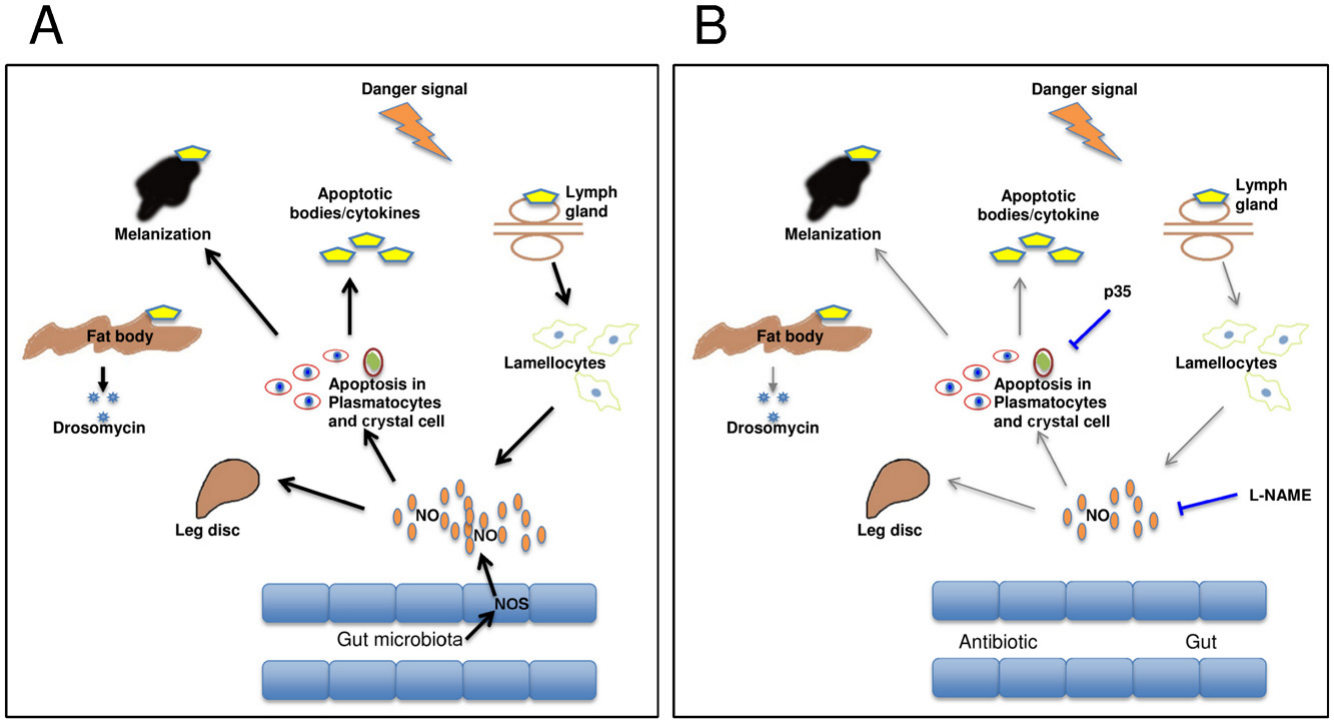
Model for the effects of apoptosis in hemocytes and the contribution of NO. (A-B) Our observations suggest that Hml-apo *Drosophila* larvae are not fully immune-deficient, but show instead a shift towards different responses (lamellocyte differentiation, appearance of melanotic masses, Toll activation) leading to developmental defects (reduction in eclosure and the defective leg phenotype). Nitric oxide acts as a key regulator explaining the rescue we observe using L-NAME. NO levels are influenced by the microbiota, which affects gut NO production (hence the rescue by antibiotics treatment and the influence of the food source) and by NO production in hemocytes, which we block using p35 (B).

The influence of antibiotics treatment and the food source pinpoints environmental differences as the most likely explanation for the discrepancies between our depletion regime and some of the previously published work, in particular those where melanization was not observed [6, 7]. To minimize genetic differences we relied on the initially published protocol, which involved activation of single apoptotic inducers and was based on the same driver and responder lines we used. An influence of the food source and the bacterial flora is in line with increasing evidence that both factors influence immune-competence in insects [30]. This includes effects on hemocyte development and maturation [31–33], which may occur in our system prior to or after apoptosis of hemocytes. Notably the gut is also the major site of expression for nitric oxide synthase [25], which corresponds with our findings that nitric oxide levels contribute to the pro-inflammatory state and ultimately to the leg defects [24]. Our tentative model (Fig 10) is based on a key role for nitric oxide as an immune regulator [34]. NO is produced in the gut but also by lamellocytes [26]. In case hemocyte depletion is complete for example by using dual proapoptotic inducers [7] the immune system will remain silent. If some plasmatocytes escape apoptosis due to the fact that the Hml driver is not expressed in all hemocytes [16, 35], the balance may shift towards activation of lamellocytes and melanization. The propensity towards immune activation depends amongst others on the NO levels, which determines whether lamellocytes are formed and melanization occurs [26] and NO levels in their turn depend partially on the food source and the resulting microbiota. Lamellocytes—once they have differentiated—are not targeted by the Hml driver and further add to the NO pool [26]. This explains why despite effective reduction in plasmatocyte numbers in Hml-apo larvae, we did not see an increase in mortality after nematode infection, since lamellocytes replace plasmatocytes upon nematode infections. In line with this we observe lamellocyte accumulation at nematode entry sites in the gut (Fig 1).

Although we experimentally induce changes in the immune status of larvae, similar scenarios may occur under natural settings. Both melanization and lamellocyte formation are strictly regulated and subject to evolutionary changes [9, 36]. For example melanization at wounds is locally enhanced due to the exposure of apoptotic markers such as phosphatidylserine on hemocytes under normal conditions in the clot [9] and lamellocyte differentiation is induced upon wounding [37]. An enhancement of parasitoid wasp egg melanization by NO–donors during encapsulation has been shown [26] and here we observe the same phenomenon in a pro-tumorous model (Fig 9). Interestingly NO also plays a key role during the development of megakaryocytes in mammals [38, 39], which is a prerequisite for functional blood clotting (Fig 1). Thus a function for NO as a signaling molecule appears to emerge as an evolutionarily conserved mechanism that regulates clot formation [34].

## Supporting Information

S1 Fig Hemocyte recruitment into circulation upon nematode infection. (A) Sessile hemocytes are present in sessile compartments in the non-infected larva. Arrows indicate RFP-positive sessile hemocytes. (B) Sessile hemocytes dispersed and migrated into circulation (hemolymph) upon nematode infection. Arrowhead indicates dispersed hemocytes in the hemolymph.
(PDF)

S2 Fig Genetic ablation of plasmatocytes and crystal cells by expressing the pro-apoptotic gene *hid*. (A-G) *hml-*Gal4 driven *hid* (transgene) expression in plasmatocytes and crystal cells eliminates them from larvae (B, D) and adults (F, G). Control larvae (A, C) and adult flies (E) where plasmatocytes and crystal cells express GFP. (H) UAS-*grim8*.*1* expression with same driver eliminated plasmatocytes and crystal cells in adults, too.
(PDF)

S3 Fig Absence of necrosis in apoptotic hemocyte population. (A-D) Control hemocytes sample (*hml-*Gal4,UAS-*eGFP*/+) and Grim- or Hid-expressing hemocytes (*hml-*Gal4,UAS-*eGFP*>UAS-*grim8*.*1* and *hml-*Gal4,UAS-*eGFP*>UAS-*hid* (L)) were analyzed with a live cell marker and apoptotic and necrotic markers (D, H, and L show merged pictures).
(PDF)

S4 Fig Coexpression of p35 with Grim in hemocytes rescues apoptosis. (A) Grim expression in hemocytes eliminates hemocytes (GFP negative). (B) Coexpression of p35 with Grim inhibits apoptosis (arrow—GFP positive hemocytes). (C-D) Flies shown in A and B are shown in bright field in C and D respectively.
(PDF)

S5 Fig Effects of hemocyte depletion during larval instars. (A) 1^st^ instar control larvae (Hml (delta)-GAL4>UAS-eGFP). Hml-(delta)-GAL4 drives UAS-eGFP expression in the early 1^st^ instar larva. (B-I) Expression of pro-apoptotic genes hid or grim efficiently removed plasmatocytes and crystal cell (leading to loss of the GFP signal) starting from the 1^st^ instar except in lymph gland. E and I are magnified sections of the rectangular areas in D and H respectively. Yellow arrows point towards GFP positive hemocytes; white arrows: lymph gland. The scale bar represents 200 μm. (J) Schematic diagram of lamellocyte counts in Hml-apo larva in different stages. A Gradual increase of lamellocytes numbers was observed from the 2^nd^ instar onwards, the highest lamellocytes were found in the late 3^rd^ larval instar.
(PDF)

S6 Fig Standard fly medium supplemented with L-arginine increases lamellocyte frequency in control larvae. A hemocyte preparation showing lamellocytes (arrow) and quantification of lamellocyte frequency after administration of the NOS substrate L-arginine are shown (*hml-*Gal4,UAS-*eGFP*>w^1118^). Lamellocyte appearance varied in *hml-*Gal4,UAS-*eGFP*>w^1118^ larvae, lamellocytes were found in one population of larvae but not in a second one (indicated as G1 and G2 respectively). The scale bars represent 50 μm.
(PDF)

S7 Fig Inhibition of NOS (using L-NAME) reduces lamellocyte numbers. Lamellocyte numbers are significantly lower in Hml-apo larvae treated with L-NAME compared to larvae treated with the enantiomer D-NAME. Mid 3^rd^ instar larvae were transferred to 50mM D-NAME- or L-NAME-containing standard fly food and hemocytes were analyzed 16 h afterwards. Administration of D-NAME or L-NAME did not alter the counts of GFP-positive hemocytes (left part).
(PDF)

S1 Table Summary of the qualitative phenotypes of the different Hid and Grim lines in combination with two hemocyte specific Gal4 lines (*hml*- and *he*- Gal4). (PDF)

S2 Table A relative comparison of the ingredients of two fly foods used in this study. The ratio of carbohydrate to protein is higher in Standard cooked fly food than in Drosophila instant medium. Other ingredients also show notable differences e.g., anti-oxidant, supplements.
(PDF)
