# Supplementary figures and images for: Apoptosis in Hemocytes Induces a Shift in Effector Mechanisms in the Drosophila Immune System and Leads to a Pro-Inflammatory State
Source: PLoS One. 2015 Aug 31;10(8):e0136593. doi: 10.1371/journal.pone.0136593 (PMC4555835; doi:10.1371/journal.pone.0136593)

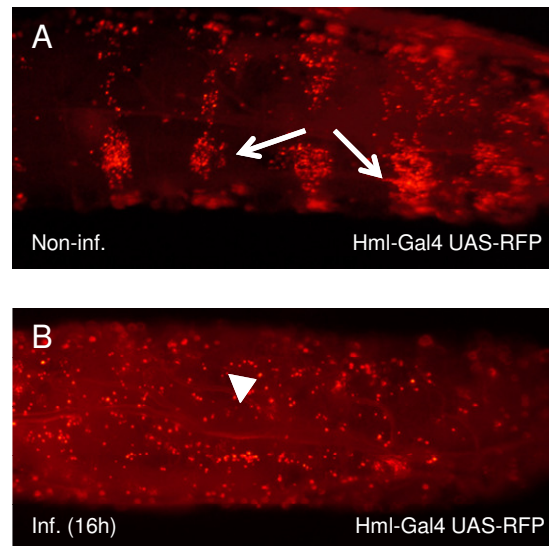

Fig. S1

Supplement: S1 Fig — (A) Sessile hemocytes are present in sessile compartments in the non-infected larva. Arrows indicate RFP-positive sessile hemocytes. (B) Sessile hemocytes dispersed and migrated into circulation (hemolymph) upon nematode infection. Arrowhead indicates dispersed hemocytes in the hemolymph. (PDF) [file pone.0136593.s001.pdf]

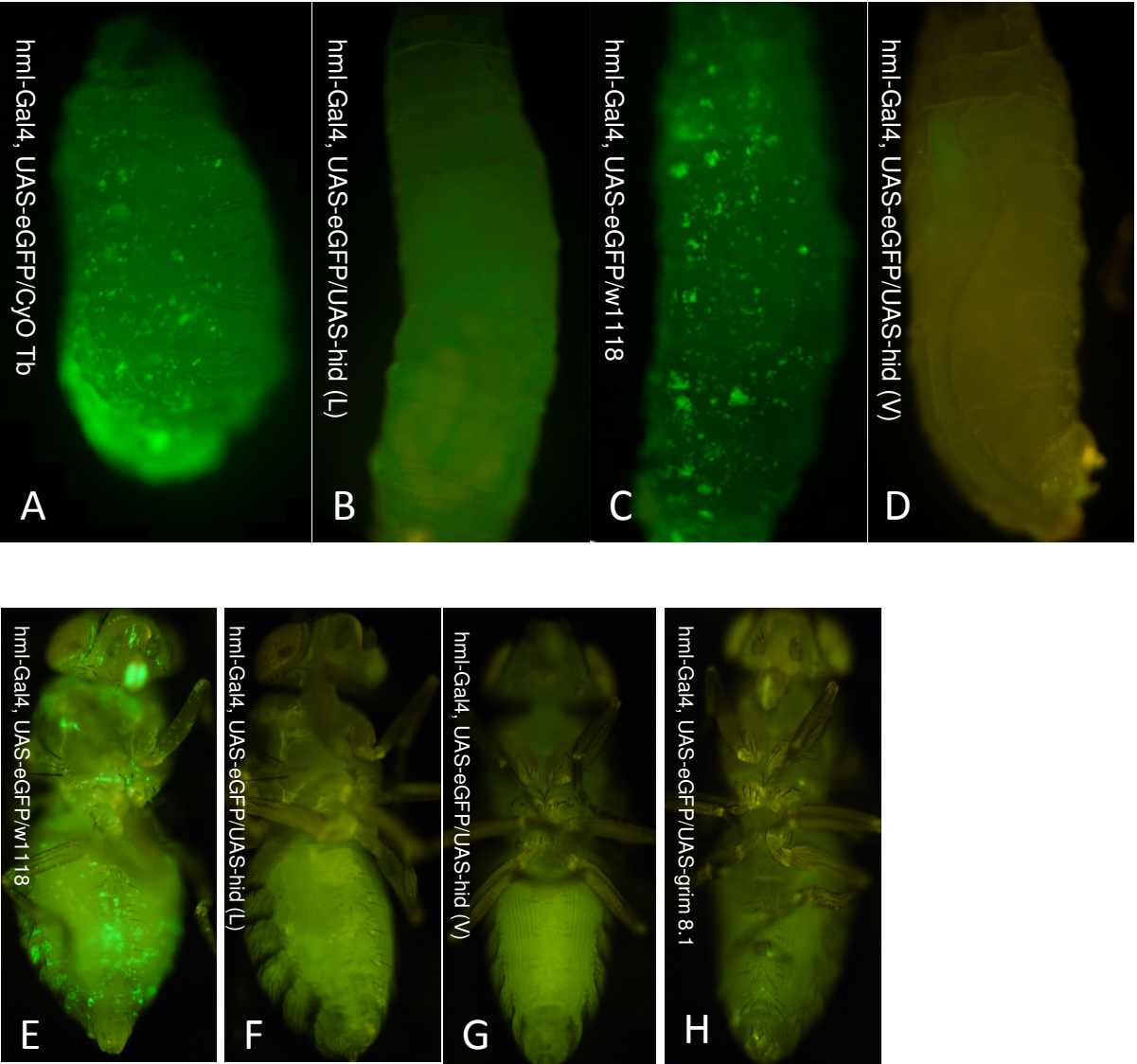

Fig. S2

Supplement: S2 Fig — (A-G) hml-Gal4 driven hid (transgene) expression in plasmatocytes and crystal cells eliminates them from larvae (B, D) and adults (F, G). Control larvae (A, C) and adult flies (E) where plasmatocytes and crystal cells express GFP. (H) UAS-grim8.1 expression with same driver eliminated plasmatocytes and crystal cells in adults, too. (PDF) [file pone.0136593.s002.pdf]

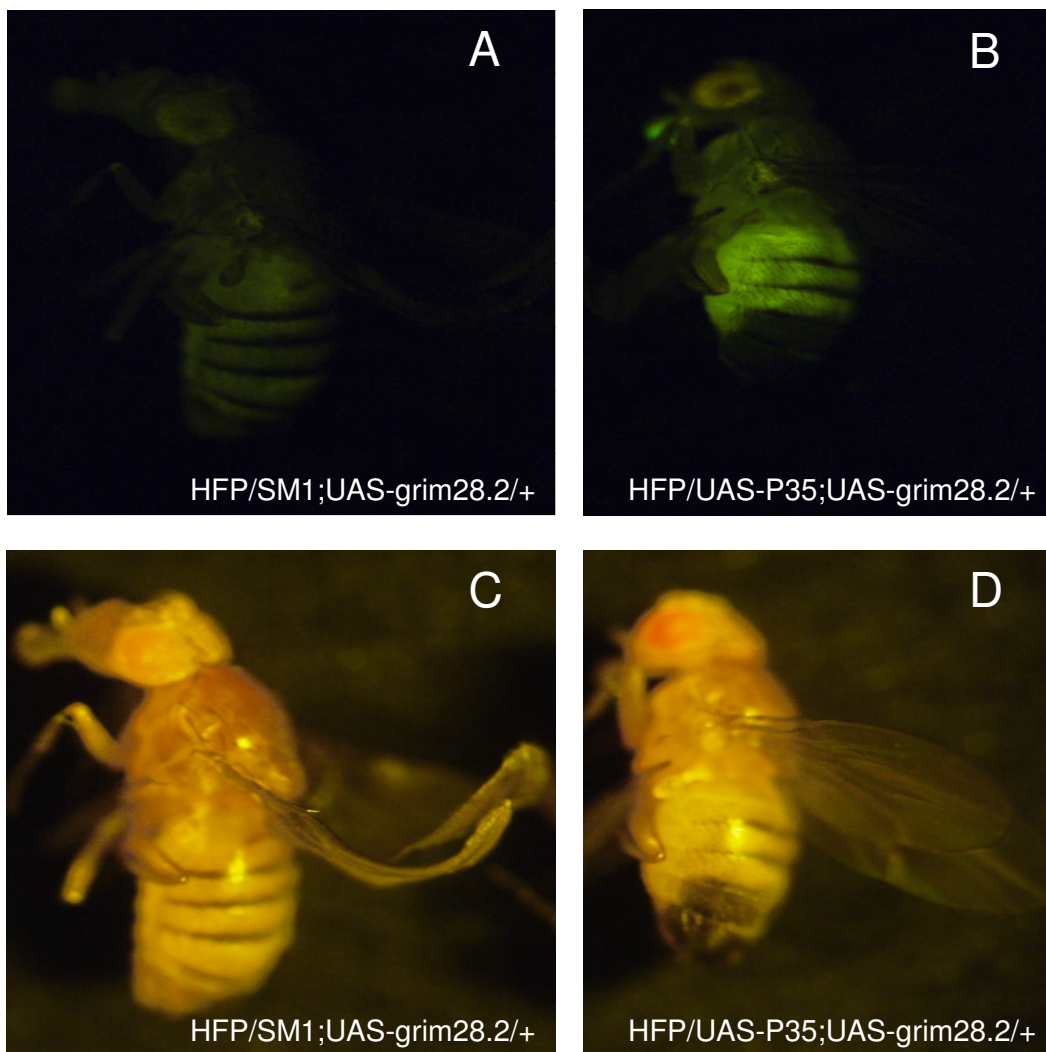

Fig. S4

Supplement: S4 Fig — (A) Grim expression in hemocytes eliminates hemocytes (GFP negative). (B) Coexpression of p35 with Grim inhibits apoptosis (arrow—GFP positive hemocytes). (C-D) Flies shown in A and B are shown in bright field in C and D respectively. (PDF) [file pone.0136593.s004.pdf]

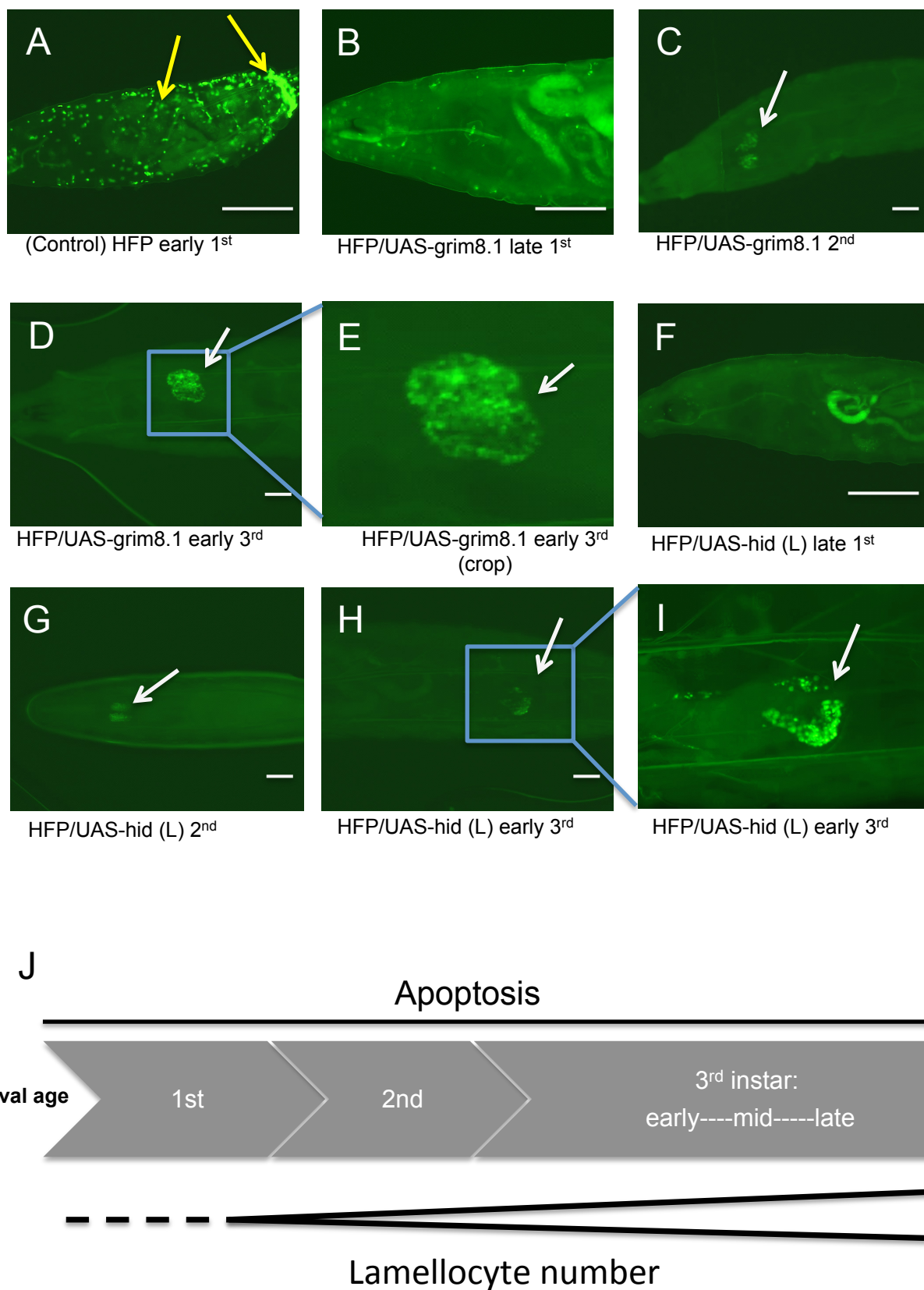

Fig. S5

Supplement: S5 Fig — (A) 1st instar control larvae (Hml (delta)-GAL4>UAS-eGFP). Hml-(delta)-GAL4 drives UAS-eGFP expression in the early 1st instar larva. (B-I) Expression of pro-apoptotic genes hid or grim efficiently removed plasmatocytes and crystal cell (leading to loss of the GFP signal) starting from the 1st instar except in lymph gland. E and I are magnified sections of the rectangular areas in D and H respectively. Yellow arrows point towards GFP positive hemocytes; white arrows: lymph gland. The scale bar represents 200 μm. (J) Schematic diagram of lamellocyte counts in Hml-apo larva in different stages. A Gradual increase of lamellocytes numbers was observed from the 2nd instar onwards, the highest lamellocytes were found in the late 3rd larval instar. (PDF) [file pone.0136593.s005.pdf]

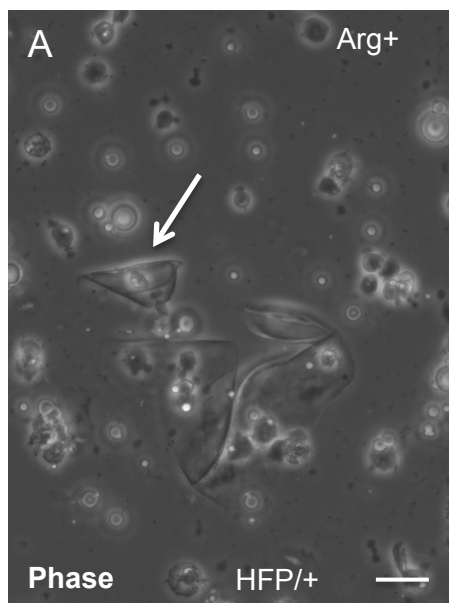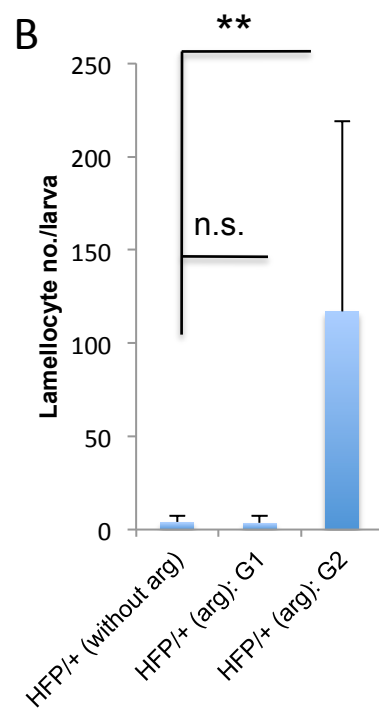

Fig. S6

Supplement: S6 Fig — A hemocyte preparation showing lamellocytes (arrow) and quantification of lamellocyte frequency after administration of the NOS substrate L-arginine are shown (hml-Gal4,UAS-eGFP>w1118). Lamellocyte appearance varied in hml-Gal4,UAS-eGFP>w1118 larvae, lamellocytes were found in one population of larvae but not in a second one (indicated as G1 and G2 respectively). The scale bars represent 50 μm. (PDF) [file pone.0136593.s006.pdf]

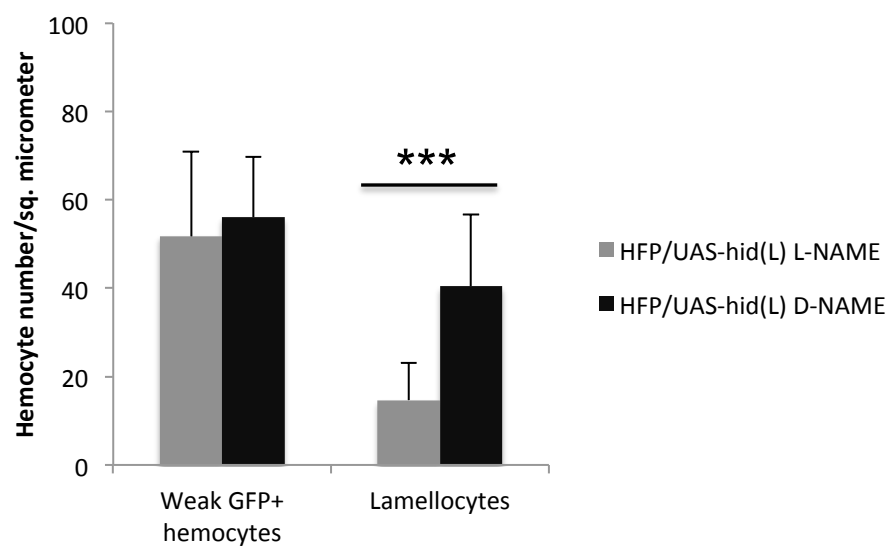

Fig. S7

Supplement: S7 Fig — Lamellocyte numbers are significantly lower in Hml-apo larvae treated with L-NAME compared to larvae treated with the enantiomer D-NAME. Mid 3rd instar larvae were transferred to 50mM D-NAME- or L-NAME-containing standard fly food and hemocytes were analyzed 16 h afterwards. Administration of D-NAME or L-NAME did not alter the counts of GFP-positive hemocytes (left part). (PDF) [file pone.0136593.s007.pdf]
