# Supplementary material for: Apoptosis in Hemocytes Induces a Shift in Effector Mechanisms in the Drosophila Immune System and Leads to a Pro-Inflammatory State
Source: PLoS One. 2015 Aug 31;10(8):e0136593. doi: 10.1371/journal.pone.0136593 (PMC4555835; doi:10.1371/journal.pone.0136593)
Supplement: S3 Fig — (A-D) Control hemocytes sample (hml-Gal4,UAS-eGFP/+) and Grim- or Hid-expressing hemocytes (hml-Gal4,UAS-eGFP>UAS-grim8.1 and hml-Gal4,UAS-eGFP>UAS-hid (L)) were analyzed with a live cell marker and apoptotic and necrotic markers (D, H, and L show merged pictures). (PDF) [file pone.0136593.s003.pdf]

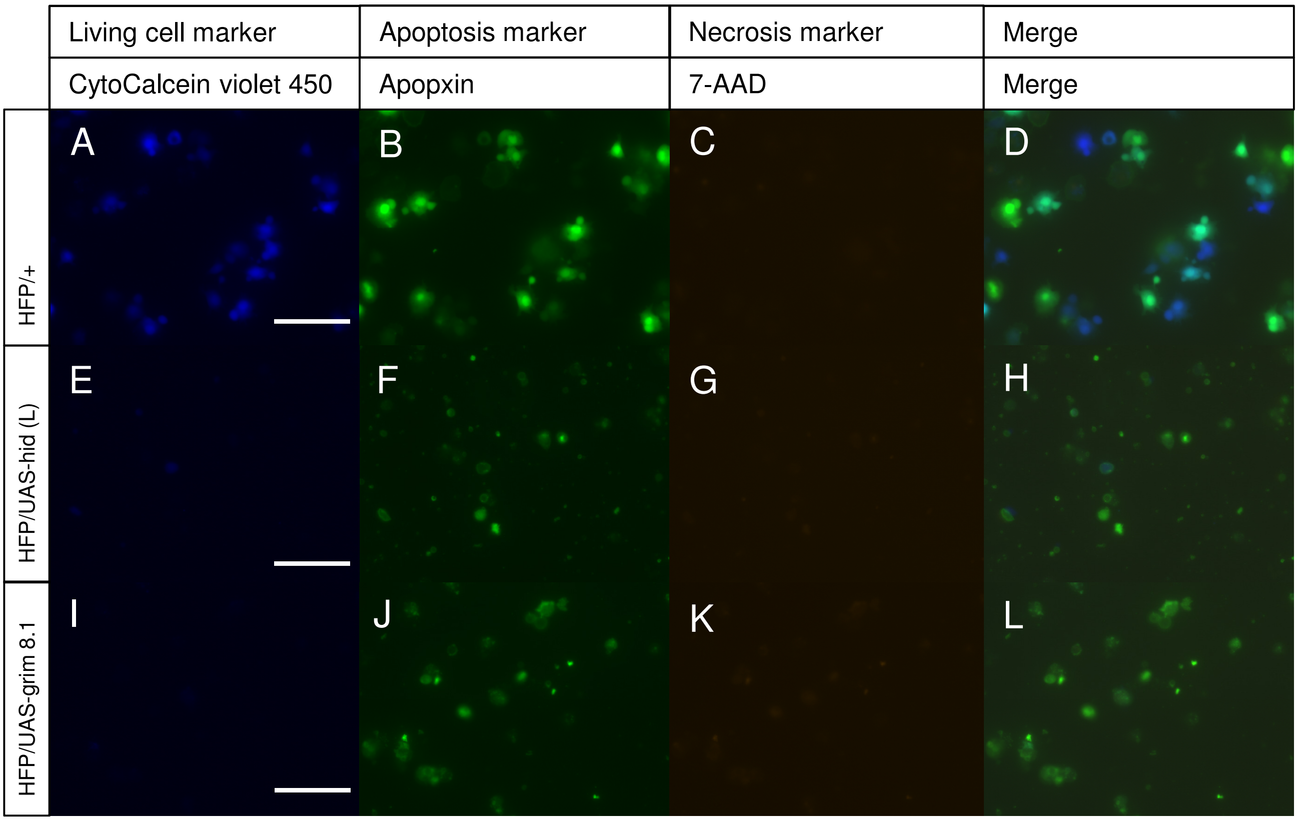

Living cell marker
CytoCalcein violet 450
Apoptosis marker
Apopxin
Necrosis marker
7-AAD
Merge
Merge
HFP/+
HFP/UAS-hid (L)
HFP/UAS-grim 8.1
A
B
C
D
E
F
G
H
I
J
K
L

Fig. S3
