# Supplementary material for: Apoptosis in Hemocytes Induces a Shift in Effector Mechanisms in the Drosophila Immune System and Leads to a Pro-Inflammatory State
Source: PLoS One. 2015 Aug 31;10(8):e0136593. doi: 10.1371/journal.pone.0136593 (PMC4555835; doi:10.1371/journal.pone.0136593)
Supplement: S1 Table — (PDF) [file pone.0136593.s008.pdf]

UAS-*hid* and UAS-*grim* lines summary table

Gal4 driver	Lines name	Survival	Larval hemocytes no. (++ -more, + -less)
hml-Gal4^{Delta}; UAS-*eGFP*	UAS-*hid* (L)	adult	+
hml-Gal4^{Delta}; UAS-*eGFP*	UAS-*hid*	adult	++
hml-Gal4^{Delta}; UAS-*eGFP*	UAS-*grim*	adult	++
hml-Gal4^{Delta}; UAS-*eGFP*	UAS-*grim8.1*	adult	++
hml-Gal4^{Delta}; UAS-*eGFP*	UAS-*grim21.1*	adult	++
he-Gal4	UAS-*hid* (L)	Embryonic lethal (EL)	
he-Gal4	UAS-*hid*	EL	
he-Gal4	UAS-*grim*	EL	

Table. S1
