# Supplementary material for: Apoptosis in Hemocytes Induces a Shift in Effector Mechanisms in the Drosophila Immune System and Leads to a Pro-Inflammatory State
Source: PLoS One. 2015 Aug 31;10(8):e0136593. doi: 10.1371/journal.pone.0136593 (PMC4555835; doi:10.1371/journal.pone.0136593)
Supplement: S2 Table — The ratio of carbohydrate to protein is higher in Standard cooked fly food than in Drosophila instant medium. Other ingredients also show notable differences e.g., anti-oxidant, supplements. (PDF) [file pone.0136593.s009.pdf]

Supplemental Table 2: Relative comparison of the ingredients between Standard Fly Food (SF) and prepared Drosophila instant medium (DIM)

No.	Ingredients	Cooked SF*	Prepared DIM**
1	Carbohydrate	8.56%	21.65%
2	Protein	0.17%	2.29%
3	Methylparaben (mold inhibitor)	0.85%	0.6%
4	Potassium	0.017%	0.26%
5	Fat	0.032%	0.17%
6	Sodium	0.028%	0.0175
7	Antioxidant	Meshed potatoes contain antioxidants, but actual percent is unknown	8.75-10 parts per million
8	Supplements	Iron, Magnesium, Copper, Calcium, Zinc, Manganese, Vitamin C.	Vitamin A, Vitamin B-6, Vitamin C, Thiamine (B-1), Riboflavin (B-2), Niacin, Calcium, Iron, Phosphorous, Magnesium
9	Water	Approx. 90%	Approx. 75%

*Ingredients estimated from the original food sources of Standard Fly Food recipe. It is cooked form.

** Carolina Biological Supply Company provided ingredients information in its powder form. No cooking, instantly made mix with water.

Table. S2
